# Electrochemical Sensing of Cadmium and Lead Ions in Water by MOF-5/PANI Composites

**DOI:** 10.3390/polym16050683

**Published:** 2024-03-02

**Authors:** Jadranka Milikić, Marjetka Savić, Aleksandra Janošević Ležaić, Biljana Šljukić, Gordana Ćirić-Marjanović

**Affiliations:** 1 Faculty of Physical Chemistry, University of Belgrade, Studentski Trg 12-16, 11158 Belgrade, Serbia; jadranka@ffh.bg.ac.rs (J.M.); gordana@ffh.bg.ac.rs (G.Ć.-M.); 2 Vinča Institute of Nuclear Science, National Institute of the Republic of Serbia, University of Belgrade, 11001 Belgrade, Serbia; metk@vin.bg.ac.rs; 3 Faculty of Pharmacy, University of Belgrade, Vojvode Stepe 450, 11221 Belgrade, Serbia; aleksandra.janosevic@pharmacy.bg.ac.rs

**Keywords:** metal-organic framework, MOF-5, polyaniline, composite, cadmium ion, lead ion, electroanalytical sensing

## Abstract

For the first time, composites of metal-organic framework MOF-5 and conjugated polymer polyaniline (PANI), (MOF-5/PANI), prepared using PANI in its conducting (emeraldine salt, ES) or nonconducting form (emeraldine base, EB) at various MOF-5 and PANI mass ratios, were evaluated as electrode materials for the electrochemical detection of cadmium (Cd^2+^) and lead (Pb^2+^) ions in aqueous solutions. Testing of individual components of composites, PANI-ES, PANI-EB, and MOF-5, was also performed for comparison. Materials are characterized by Raman spectroscopy, scanning electron microscopy (SEM) and dynamic light scattering (DLS), and their electrochemical behavior was discussed in terms of their zeta potential, structural, morphology, and textural properties. All examined composites showed high electrocatalytic activity for the oxidation of Cd and Pb to Cd^2+^ and Pb^2+^, respectively. The MOF/EB-1 composite (71.0 wt.% MOF-5) gave the highest oxidation currents during both individual and simultaneous detection of two heavy metal ions. Current densities recorded with MOF/EB-1 were also higher than those of its individual components, reflecting the synergistic effect where MOF-5 offers high surface area for two heavy metals adsorption and PANI offers a network for electron transfer during metals’ subsequent oxidation. Limits of detection using MOF/EB-1 electrode for Cd^2+^ and Pb^2+^ sensing were found to be as low as 0.077 ppm and 0.033 ppm, respectively. Moreover, the well-defined and intense peaks of Cd oxidation to Cd^2+^ and somewhat lower peaks of Pb oxidation to Pb^2+^ were observed at voltammograms obtained for the Danube River as a real sample with no pretreatment, which implies that herein tested MOF-5/PANI electrodes could be used as electrochemical sensors for the detection of heavy metal ions in the real water samples.

## 1. Introduction

Monitoring of heavy metal ions such as cadmium (Cd^2+^) and lead (Pb^2+^) ions is an imperative due to their high toxicity, even in low concentrations, and harmful effect on human health and the environment [1,2]. The development of industry and agriculture has lead to an increasing amount of heavy metal ions in surface and subsurface waters [3]; namely, wastewater contains these heavy metal ions as a product of battery manufacturing, paint manufacturing, metal plating, pesticides, etc. [3]. Cd^2+^ and Pb^2+^ are non-biodegradable, and they cannot be easily eliminated; thus, their presence could change biological cell function and cause blood, brain, and nervous systems problems in humans, animals, and plants [1,4]. Excretion of these metals from the body is possible, but notably slower than the extraction of other molecules [2,4]. Therefore, the permissible concentrations of Cd^2+^ and Pb^2+^ ions in drinking water set by the World Health Organization (WHO) are as low as 10 and 3 ppb (i.e., 0.01 and 0.003 ppm), respectively [5,6].

Atomic absorption spectroscopy (AAS), X-ray fluorescence spectrometry (XRF), inductively coupled plasma mass spectroscopy (ICP-MS), inductively coupled plasma-optical emission spectrometry (ICP-OES), and atomic fluorescence spectroscopy (AFS) are conventional analytical methods for Cd^2+^ and Pb^2+^ ions detection, all involving expensive equipment and complex operations [1,7]. Electroanalytical methods such as anodic stripping voltammetry (ASV), are simple, fast, low-cost, and highly sensitive in terms of heavy metal ions sensing, also offering the benefit of portability [8]. During recent decades, the research for suitable electrode materials for the electrochemical sensing of heavy metal ions in aqueous media was intense, and this topic is still relevant and challenging. These materials should possess desirable properties, such as large electroactive surface area, high porosity, electrical conductivity, stability, as well as high sensitivity and selectivity toward particular heavy metal ions. Various classes of materials were explored for this purpose, among which carbon materials, metal oxides, conducting polymers, metal-organic frameworks and their composites have received great attention.

Metal-organic frameworks (MOFs) are highly crystalline, porous, and large surface area materials that have particular properties originating in their specific structures made of flexible organic and rigid inorganic parts. They are also called coordination polymers since they are formed by the coordination of metal ions and organic bridging linkers [9]. MOFs have broad applicability in gas storage, energy storage, catalysis, etc. One of the first discovered MOFs was MOF-5, which consists of [Zn_4_O]^6+^ units connected to linear 1,4-benzenedicarboxylate (BDC) structural units (organic linker), forming a porous framework with the composition Zn_4_O(BDC)_3_ [10]. It has desirable properties, such as high crystalline (cubic) structure, microporosity, and large pore volume, but with two drawbacks: low electrical conductivity and high influence of moisture on its stability [9]. On the other hand, conducting polymers such as polyaniline (PANI) exhibit high electrical conductivity and redox activity, with good electrochemical and environmental stability and other desirable properties for various applications, but suffer from low surface areas [9,11].

Because of their attractive structure and suitable properties, MOFs electrodes and MOFs modified carbon-based electrodes were used for electrochemical sensing of several analytes [12], such as heavy metals [12,13,14,15,16,17], nitrogen compounds [18], glucose [12], dopamine [19], ascorbic acid [12], etc. For instance, the Zr-based MOF UiO-66, as well as its carbonized form (CUiO-66), were examined as electrodes for Cd^2+^ and Pb^2+^ ions detection by square wave ASV [16]. These electrodes showed good stability and selectivity with the limit of detection (LOD) of 1.16 ppb (0.00116 ppm) for Cd^2+^ and 1.14 ppb (0.00114 ppm) for Pb^2+^ [16]. MOF-5-modified carbon paste electrodes [17] as well as nanocomposite of manganese-terephthalic acid MOF withsingle-walled carbon nanotubes (Mn(TPA)-SWCNTs) [14] showed good sensitivity and low LOD for determination of Pb^2+^. MOFs cauliflower-like MIL-100(Cr) was tested for simultaneous heavy metal ions detection in aqueous media, and it showed LOD of 0.1 and 2.9 ppb (0.0001 and 0.0029 ppm) for Cd^2+^ and Pb^2+^, respectively [20]. Cd^2+^ detection was investigated using MOF TMU-16-NH_2_ ([Zn_2_(NH_2_-BDC)_2_(4-bpdh)]·3DMF) electrodes by differential pulse voltammetry [21]. This electrode material showed low LOD, good response in the real sample, and good sensitivity [21]. The authors explained that the oxidation of Cd on TMU-16-NH_2_ modified graphene/carbon paste electrodes depends on the interactions between Cd and amine groups [21].

On the other hand, electrodes based on conducting polymer polyaniline (PANI) also present attractive sensors for heavy metals due to their high specific capacitance, easy synthesis, and low cost [22,23]. An electrode with self-doped PANI nanofibers/mesoporous carbon nitride (MCN) deposited on a glassy carbon was tested for simultaneous determination of trace amounts of Cd^2+^ and Pb^2+^ ions by square wave ASV, and showed LOD of 0.7 ppb (0.0007 ppm) for Cd^2+^ and 0.2 ppb (ppm) for Pb^2+^ ions [24]. PANI/graphene nanocomposite electrode was studied as an electrochemical sensor for simultaneous detection of Cd^2+^, Pb^2+^ and Zn^2+^ ions with detection limits of 0.1 ppb (0.0001 ppm) for both Cd^2+^ and Pb^2+^ [25]. Nanocomposites based on PANI, modified with ethylenediaminetetraacetic acid (EDTA) chelating ligand and on single-walled carbon nanotubes (SWCNTs) (PANI/SWNCTs) [22] and PANI nanowire arrays (PANI-NWA) [23], present high active sensors for simultaneous and selective detection of heavy metal ions. The composite of bimetallic CoCu-MOF and PANI, synthesized by a one-step in situ polymerization method, showed activity for simultaneous electrochemical detection of heavy metal ions with good linearity in the range 5–100 ppb (0.005–0.1 ppm) and 7.5–150 ppb (0.0075–0.15 ppm) for Pb^2+^ and Cd^2+^, respectively [26].

Recently, we synthesized electroconducting MOF-5/PANI composites with high specific surface areas [9] which overcome the shortcomings of the individual components, such as the low surface area of PANI and poor electrical conductivity of MOF-5. Therefore, we supposed that the composites would behave better than their starting individual components (PANI and MOF-5) in various applications, and we continued our research in the direction of their environmental applications as materials for detection of water pollutants. Due to their large specific surface area, we believed that the MOF-5/PANI composites would be suitable as electrode materials in electroanalysis. To the best of our knowledge, composites of MOF-5 and PANI have not yet been explored as electrochemical sensors for heavy metal ions. In this work, for the first time six different MOF-5/PANI composites were evaluated as sensors for the electroanalytical detection of Cd^2+^ and Pb^2+^ ions in water samples. Both individual and simultaneous detection of these two heavy metal ions was examined by using the ASV technique. The sensing behaviour of composites was compared with that of their starting individual components, PANI and MOF-5, under the same conditions. Differences in material’s electrochemical performance are discussed in terms of their structure, morphology and zeta-potential, investigated by Raman spectroscopy, scanning electron microscopy (SEM) and dynamic light scattering (DLS), respectively, and related also with their reported textural properties (specific surface area, pore volume) [9]. Simultaneous electrochemical detection of Cd^2+^ and Pb^2+^ ions by MOF-5/PANI composites and their precursor components was also investigated using the real water sample from Danube River, without pretreatment.

## 2. Materials and Methods

Benzene-1,4-dicarboxylic acid (terephtalic acid, H_2_BDC, p.a. > 98%, Biochem Chemopharma Cosne-Cours-sur-Loire, France), zinc acetate dihydrate (p.a. > 99%, Centrohem, Stara Pazova, Serbia), dimethylformamide (DMF, p.a. > 99.5%, Centrohem, Stara Pazova, Serbia), chloroform (p.a. > 99%, Centrohem, Stara Pazova, Serbia), HCl (p.a. > 36.5%, Centrohem, Stara Pazova, Serbia), ammoniumperoxydisulfate (APS, p.a. > 98%, Centrohem, Stara Pazova, Serbia), ethanol (p.a. 96%, Centrohem, Stara Pazova, Serbia) and ammonium hydroxide (p.a. 25%, Centrohem, Stara Pazova, Serbia) were used as received. Aniline (p.a. > 99.5%, Centrohem, Stara Pazova, Serbia) was distilled under reduced pressure and stored under argon prior to use. Vulcan^®^ XC72 (Cabot Corporation, Boston, MA, USA), Nafion™ 117 (Sigma-Aldrich, St. Louis, MO, USA), 96% sulfuric acid (Suprapur^®^, Merck, Darmstadt, Germany), lead(II) nitrate (p.a. > 99%, Centrohem, Stara Pazova, Serbia), cadmium sulfate 8/3-hydrate (Thomas Tyrer Co. Ltd. Stratford, London, UK), sodium chloride (Zorka Šabac, Serbia).

MOF-5 component of MOF-5/PANI composites was synthesized by the procedure developed by Yaghi’s group [27] and modified by Savić Biserčić et al. [10] so that anhydrous zinc acetate replaced zinc acetate dihydrate, with the addition of the optimal amount of water (mole ratio H_2_O/Zn^2+^ = 0.5).

Composites MOF-5/PANI were prepared by the procedures reported in ref. [9] using different MOF-5 to PANI mass ratios. The first series of composites, MOF/ES, was prepared by using PANI in its conducting emeraldine salt (ES) form, PANI-ES, which was synthesized by the oxidative polymerization of aniline in the presence of HCl. The second series of composites, MOF/EB, was prepared using PANI in nonconducting, emeraldine base (EB) form, and PANI-EB, synthesized in water without added acid and deprotonated. For the MOF/ES series, a mechano–chemical synthetic approach was used, with measured amounts of MOF-5 and PANI-ES being vigorously crashed and mixed in a mortar with pestle in the presence of small amount of chloroform until its complete evaporation. Composites of MOF/EB series were prepared by mixing dissolved parts of PANI-EB in DMF with MOF-5. Composites of MOF/ES series contained 25.2, 50.7 and 77.2 wt.% of MOF-5 [9] and are denoted MOF/ES-1, MOF/ES-2 and MOF/ES-3, respectively. Composites of the MOF/EB series contained 71.0, 77.4 and 89.0 wt.% of MOF-5 [9] and are abbreviated as MOF/EB-1, MOF/EB-2 and MOF-EB-3, respectively.

Raman spectra of studied materials in powder state were recorded with DXR Raman Microscope (Thermo Scientific) equipped with a research optical microscope and a CCD detector. The spectra were recorded using a diode-pumped solid-state high-brightness laser with an excitation wavelength (λ_exc_) of 532 nm, the laser power on the sample of 10 mW (for MOF-5) and 1 mW (for MOF-5/PANI composites), 10 s exposure time and 10 exposures per spectrum. The powdered sample was placed on an X–Y motorized sample stage and the laser beam was focused on the sample using an objective magnification of ×50. The scattered light was analyzed by the spectrograph with 900 lines/mm grating.

The investigated materials were characterized in terms of zeta potential by a dynamic light scattering (DLS) technique, using Zetasizer Nano ZS ZEN 3600 (Malvern). For these measurements, 0.5 mg of the sample (MOF-5/PANI composite, MOF-5 or PANI) was dispersed in 10 mL of 20 mM H_2_SO_4_ + 30 mM KCl (pH ≈ 3). After homogenization in an ultrasonic bath for 10 min, the zeta potential of dispersed particles was measured and the results were expressed as the mean value of 3 measurements. All measurements were performed at 25 °C.

A scanning electron microscope (SEM) PhenomProX SEM-EDX (Phenom, Rotterdam, The Netherlands) was used to investigate the morphology of the studied materials.

Catalytic inks were prepared by mixing synthesized material (MOF-5/PANI composite, MOF-5 or PANI, 3 mg), Vulcan (0.6 mg), ethanol (450 µL), and Nafion (0.5%, 20 µL) in an ultrasonic bath for 1 h.

PalmSense EmStat3 Blue Potentiostat and a glass cell of 25 mL volume with a three-electrode system were used for all electrochemical measurements where a saturated calomel electrode (SCE, HI5412, Hanna Instruments) and graphite rod (Sigma–Aldrich, 99.995 wt.%) were set as reference and counter electrode, respectively. Catalytic ink (15.3 µL) of the studied material was drop-casted on a conductive support (0.3 cm^2^) and set as a working electrode. In all experiments, 20 mM H_2_SO_4_ + 30 mM KCl (pH ~ 3) was used as a supporting electrolyte.

Anodic stripping voltammetry involved the accumulation step at −1.3 V for 120 s, followed by the stripping step changing the potential from −1.3 to 0 V at a rate of 50 mV s^−1^. Both individual and simultaneous sensing of Cd^2+^ and Pb^2+^ were performed in 100 µM solution of the corresponding metal in the supporting electrolyte. The limit of detection was determined in a concentration range from 0.7 to 1.5 µM for Cd^2+^ and from 0.7 to 1.2 µM for Pb^2+^ in six and nine increments, and with the accumulation step at −1.3 V for 300 s.

All composites were also examined as electrode materials for simultaneous Cd^2+^ and Pb^2+^ ions detection in a real water sample (i.e., the Danube River sample) with no pretreatment.

## 3. Results

### 3.1. Characterization of Materials

#### 3.1.1. Molecular Structure and Morphology—Raman Spectroscopy and SEM

**Figure 1:**
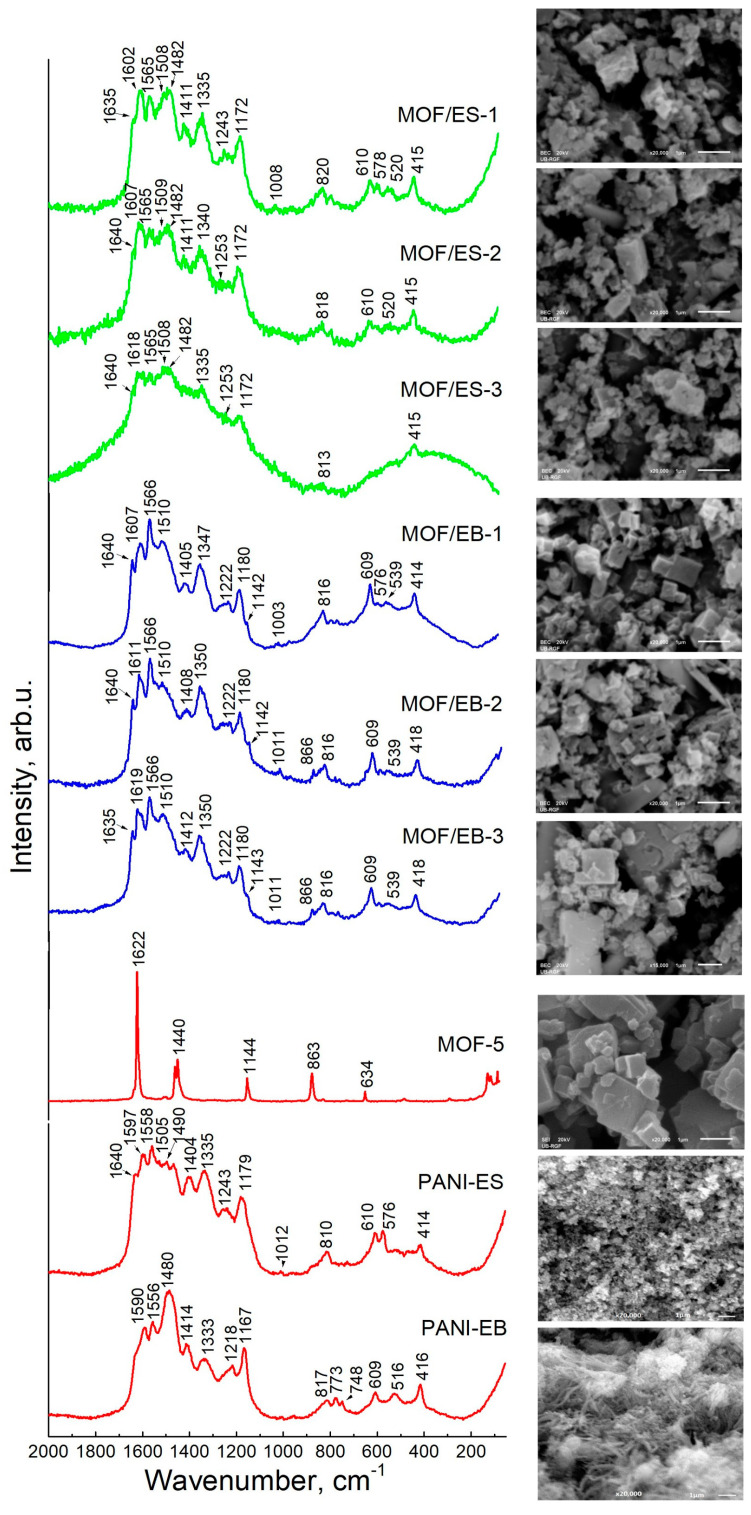
Raman spectra recorded at λ_exc_ =532 nm (**left**) and SEM images (**right**) of MOF-5/PANI composites and their starting components MOF-5, PANI-ES and PANI-EB. The scale bar on SEM images is 1 μm.

Raman spectra and SEM images of MOF-5/PANI composites and their individual precursor components are shown in Figure 1.

The Raman spectra confirm the presence of both PANI and MOF-5 components in the MOF-5/PANI composites by their characteristic bands. The strong bands observed at 1602–1618 cm^−1^ and 1607–1619 cm^−1^ in the spectra of MOF-ES and MOF/EB composites, respectively, can be attributed to the contributions of vibrations in both MOF-5 and PANI. This corresponds to the very strong band seen at 1622 cm^−1^ in the spectrum of bare MOF-5 (Figure 1), assigned to the C=C stretching vibration of the benzene ring (ν(C=C)_B_) in BDC linker [28,29], as well as to the characteristic PANI band seen at c.a. 1597 and 1590 cm^−1^ in the spectra of PANI-ES and PANI-EB, respectively, assigned to ν(C=C)_Q_/ν(C~C)_SQ_ and ν(C=C)_Q_ vibrations, respectively (Q and SQ denote quinonoid and semiquinonoid ring, respectively) [30]. This band shifts to higher wavenumbers with increasing the content of MOF-5 in the composites, approaching the position of 1622 cm^−1^ found in the spectrum of pure MOF-5. In the spectra of composites with high MOF-5 content, MOF/EB-2 and MOF/EB-3, an additional band characteristic of MOF-5 is seen at 866 cm^−1^, which corresponds well with the band observed at 863 cm^−1^ in the spectrum of bare MOF-5, and it is assigned to the out-of-plane C–H deformation vibration of the benzene ring in BDC linker [29]. For all MOF/EB composites, we observe another band assigned to MOF-5 at c.a. 1142 cm^−1^, which corresponds to the band at 1144 cm^−1^ in the spectrum of pure MOF-5 and can be attributed to the in-plane C–H deformation vibration of the benzene ring in BDC [31,32]. The remaining two bands of MOF-5 (at 1440 and 634 cm^−1^ in the spectrum of bare MOF-5) are not seen in the spectra of composites as being overlapped by the stronger bands of the PANI component. Furthermore, beyond the band at c.a. 1600–1620 cm^−1^, other bands assigned to the PANI part in the composites are seen at c.a. 1640, 1565, 1482, 1411, 1335, 1253, 1172, 820, 610 cm^−1^ in the spectra of MOF/ES and at 1640,1566, 1510, 1408, 1350, 1222, 1180, 816, 609 cm^−1^ in the spectra of MOF/EB. The strong band seen at 1335 cm^−1^ in the spectrum of PANI-ES is assigned to ν(C–N^+•^) vibrations in polaron structures. This band represents a common indicator of a PANI part’s good electrical conductivity, together with the band at c.a. 1170 cm^−1^ due to in-plane deformation δ(C–H) vibration of the SQ rings [30]. In the spectra of MOF/ES composites, we see additional strong ν(C–N^+•^) bands at similar positions of 1335 and 1340 cm^−1^, and their intensity decreases with decreasing PANI content in composites. The band observed at 1333 cm^−1^ in the spectrum of PANI-EB is much weaker. However, unexpectedly, in the spectra of MOF/EB composites, the band attributable to ν(C–N^+•^) vibration at 1350 cm^−1^ is noticeably stronger than the band at 1333 cm^−1^ in the spectrum of PANI-EB. This feature could be explained by partial resistance to dedoping of PANI-ES used for the preparation of MOF/EB composites, leading to the presence of residual polarons (semiquinone radicals, –NH^+•^), which are probably mainly localized. Similar behavior was observed and discussed previously for PANI nanotubes/nanorods synthesized in water without added acid [30,33]. An additional reason could be a partial excitation of EB to ES structures (‘redoping’), that can be induced by the incident laser radiation during recording the Raman spectra [34] and promoted by the presence of a MOF-5 component, leading to an enhancement of the ν(C–N^+•^) band. The importance of MOF-5 in this process of transforming EB to ES structures is supported by the literature data on the interfacial charge transfer processes from the photoexcited MOF-5 to the surface adsorbates, i.e., on the photoinduced one-electron oxidation reactions of organic compounds (substrates), such as aromatic sulfides and amines on MOF-5, upon the photoexcitation of MOF-5 [35]. The very strong band at 1480 cm^−1^, characteristic of nonconducting PANI base form and attributed to ν(C=N)_Q_ vibrations, is observed in the spectrum of PANI-EB. However, interestingly, in the spectra of MOF/EB, instead of the band at 1480 cm^−1^, the band is seen at 1510 cm^−1^, which is a position characteristic for the ES form with assignation to δ(N–H)/ν(C=N)_Q_ vibrations [30,33]. This feature additionally supports the assumption about partial redoping of the PANI-EB part in the composites, influenced by the surrounding MOF-5. The spectra of MOF/ES samples show the strong PANI band with two maxima at c.a. 1510 and 1480 cm^−1^. The samples of both MOF/ES and MOF/EB series show the bands at c.a. 1640 and 1410 cm^−1^ assignable to non-standard PANI units, such as substituted phenazine-type, formed due to branching and intramolecular oxidative cyclization reactions [30].

SEM images in Figure 1. have shown differences in the morphology of the studied materials. Pure MOF-5 exhibits pure cubic morphology [9], while PANI-ES and PANI-EB have granular and predominately 1-D nanostructured (nanotubes, nanorods) morphologies, respectively. In MOF/EB composites, the 1-D nanostructured morphology of the PANI-EB precursor is lost, probably due to the effect of the DMF solvent during the syntheses, while cubic morphology originating from MOF-5 is preserved to a large extent. The most regular morphology is observed for the MOF/EB-1 sample, with prevalently cubic particles of submicron size. The samples MOF/EB-2 and MOF/EB-3 contain larger content of differently shaped particles, in addition to cubic ones, with sizes in the submicron and micron range. The composites of the MOF/ES series also contain cube-like particles, but mainly with flattened edges, in addition to particles of different shapes of submicron and micron size.

#### 3.1.2. Zeta Potential by DLS

The values of zeta potential of the studied MOF-5/PANI composites and their precursor components dispersed in 20 mM H_2_SO_4_ + 30 mM KCl, determined by DLS, are shown in Table 1. For the pristine MOF-5 sample, a negative value of the zeta potential close to zero (−0.12 mV) was measured, indicating poor stability of its dispersion and a tendency towards agglomeration. All other studied materials have positive zeta potential values in the range ca. 7–15 mV, implying better stability in their dispersions. The composites of the MOF/EB series have higher zeta potential values (and thus better dispersion stability) then MOF/ES composites, with the highest value of 14.93 mV measured for the MOF/EB-1 sample. Within each series, the zeta potential decreases while increasing the amount of MOF-5 in the composite. It also can be observed that zeta potential values for MOF/EB series composites (14.93–11.77 mV) are lower than the value measured for their precursor PANI-EB (15.2 mV), and, similarly, zeta potential values for the MOF/ES series (11.73–6.92 mV) are lower than the value for their precursor PANI-ES (13.14 mV). These results imply that the presence of MOF-5 in the composites generally led to a decrease in their zeta potential compared to values for the corresponding PANI precursor and to more pronounced particle agglomeration.

### 3.2. Electrochemical Characterization

**Figure 2:**
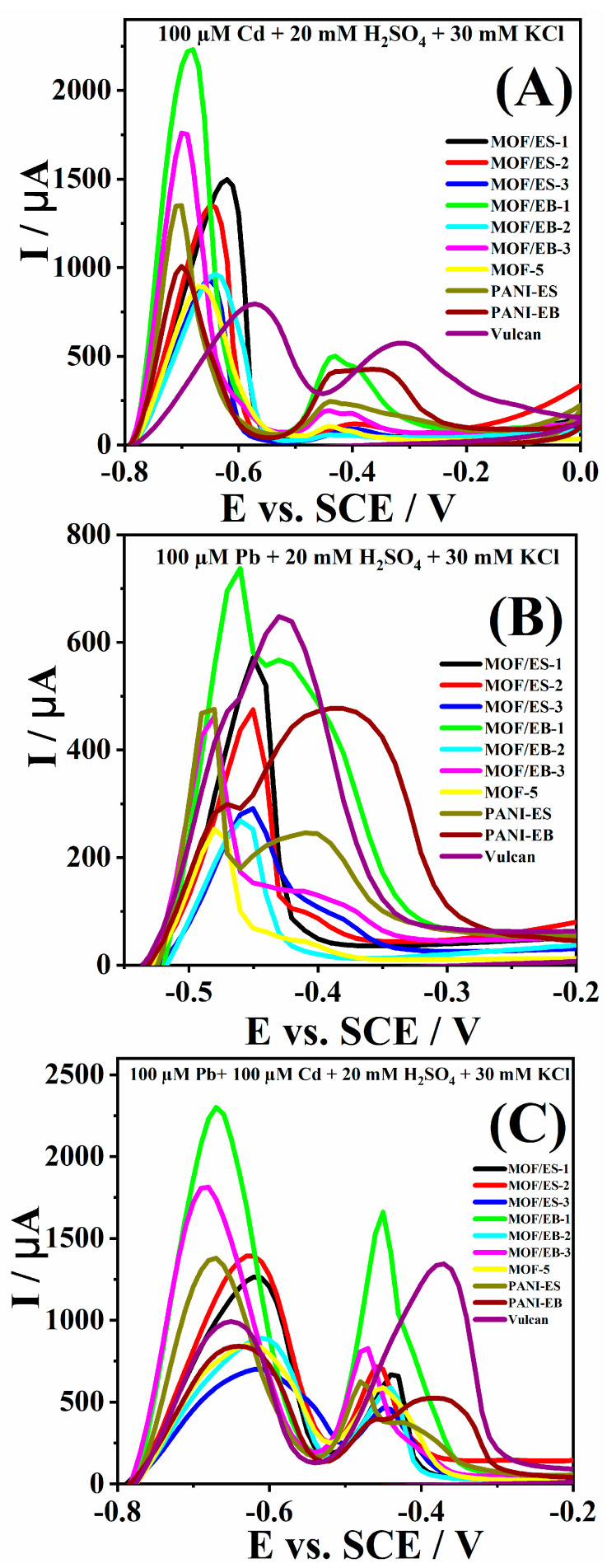
Voltammograms of six MOF-5/PANI composites, PANI-ES, PANI-EB, MOF-5 and Vulcan electrodes in 100 µM Cd^2+^ + 20 mM H_2_SO_4_ + 30 mM KCl (**A**), 100 µM Pb^2+^ + 20 mM H_2_SO_4_ + 30 mM KCl (**B**), and 100 µM Cd^2+^+ 100 µM Pb^2+^ + 20 mM H_2_SO_4_ + 30 mM KCl (**C**) at a scan rate of 50 mV s^−1^.

Figure S1 shows voltammograms of six MOF-5/PANI composites and their individual components, PANI-ES, PANI-EB and MOF-5, in the supporting electrolyte and in 100 µM Cd^2+^ solution. A well-defined peak in the potential range from −0.7 to −0.6 V corresponding to the oxidation of Cd to Cd^2+^ is observed in 100 µM Cd^2+^ solution (Figure 2A and Figure S1) [16,36,37,38]. An additional small oxidation peak appeared in the potential range from −0.5 to −0.4 V (Figure S1), most likely due to the oxidation of hydrogen [38,39]. It should be noted that no oxidation peaks were observed in the supporting electrolyte without analyte. Comparative analysis of peak currents reveals the highest current of 2.235 mA at −0.68 V during the oxidation of Cd at the MOF/EB-1 electrode, followed by the MOF/EB-3 electrode with an oxidation current value of 1.765 mA at −0.70 V (Figure 2A). On the other hand, MOF/EB-2, MOF/ES-3, and MOF-5 gave the lowest oxidation currents in the −0.64 to −0.67 V range (Table 1). As Vulcan was added during the electrodes preparation to further increase the electric conductivity of the composites, its response to the Cd^2+^ presence was also recorded. A peak corresponding to Cd oxidation at Vulcan appeared at −0.57 V with a current value of 0.797 mA (Figure S1J), i.e., ca. three times lower than the peak current recorded in the case of the MOF/EB-1 composite, indicating significantly higher activity for Cd^2+^ ions detection of the herein tested composite compared to Vulcan. As peak current is directly proportional to the amount of analyte adsorbed on the electrode surface during the accumulation period, the highest peak current in the case of MOF/EB-1 might be due to its high specific surface area of 1642.8 m^2^ g^−1^ [9]. However, the synergistic effects of other factors must be considered since the other two samples in the series with PANI-EB also have high surface areas [9]. The effect of materials’ morphology seems to play an important role in their electrochemical behavior, as composite MOF/EB-1 contains the smallest particles (submicron sized) and shows the most ordered morphology among the composites with predominantly cube shaped particles (Figure 1). Thus, accumulation of the largest amount of Cd in the case of MOF/EB-1 is facilitated due to interplay between its most regular morphology among composites and high specific surface area. Also, its highest zeta potential (indicative of fewer tendencies toward the aggregation of particles (Table 1)) and large micropore volume [9] can be additional favorable factors contributing to its highest electrocatalytic activity.

Furthermore, an intensive anodic peak was observed between −0.39 and −0.48 V for all tested materials in 100 µM Pb^2+^ solution (Figure S2), corresponding to the oxidation of Pb to Pb^2+^ [36,37,38]. The MOF/EB-1 electrode again showed the highest activity for the oxidation of Pb, with the highest current of 0.733 mA at −0.46 V, whereas MOF/ES-1 gave a somewhat lower oxidation current of 0.574 mA at −0.45 V (Figure S2, Table 2). Much lower peak currents of 0.251, 0.263, and 0.286 mA were noticed at voltammograms of MOF-5, MOF/EB-2, and MOF/ES-3, respectively, which implies that these three materials have the lowest activity for Pd^2+^ ion detection. The results showed that the composites with the lowest content of MOF-5 in each series, MOF/EB-1 and MOF/ES-1, showed the best response to Pb^2+^ presence among composites, also better than that of bare MOF-5 and PANI. These two composites have the highest zeta potential values within their series, and thus the best dispersibility. Other relevant factors regarding the best electrochemical behaviour of MOF/EB-1 can be found in the material’s morphology and textural characteristics, similar to the explanation offered for Cd^2+^ detection. Synergism between PANI and MOF-5 in composites is important, particularly the enlargement of material’s specific surface area due to MOF-5 and the increase in electrical conductivity enabled by the PANI component. Based on well-known acid-base (doping-dedoping) transitions of PANI [11], we suppose that, in the presence of an acidic supporting electrolyte (20 mM H_2_SO_4_ + 30 mM KCl of pH ≈ 3), the PANI-EB form in composites has been transformed into a conducting PANI-ES form, and thus under applied experimental conditions in the electrochemical cell MOF/EB and MOF/ES, composites behave similarly in terms of their electrical conductivity.

It should be mentioned that all composites were found to be stable over time, giving steady and reproducible responses to two heavy metal ions’ presence within a period of several months.

Figure 2C and Figure S3 show voltammograms of all tested materials for the simultaneous detection of Cd^2+^ and Pb^2+^ ions. These voltammograms presented the same trend of oxidation currents as during the individual detection of Cd^2+^ and Pb^2+^ ions. MOF/EB-1 composite gave the highest oxidation currents for both heavy metal ions, followed by the MOF/EB-3 composite. It should be noted that the peaks corresponding to the oxidation of Cd and Pb to Cd^2+^ and Pb^2+^, respectively, were observed with good resolution and that the presence of the other ion did not affect the composites’ response. Namely, electrode materials often show higher adsorption capacity and selectivity for Pb ions compared to other heavy metals in the presence of small amounts of heavy metal ions [40]. Thus, simultaneous electroanalytical sensing of Cd^2+^ and Pb^2+^ ions is very often hampered by the higher affinity of functional groups of the electrode materials for Pb^2+^ ions compared to Cd^2+^ ions during the accumulation step [15,41,42]. This results in a low/no oxidation peak corresponding to the Cd^2+^ ions’ presence [15].

**Figure 3:**
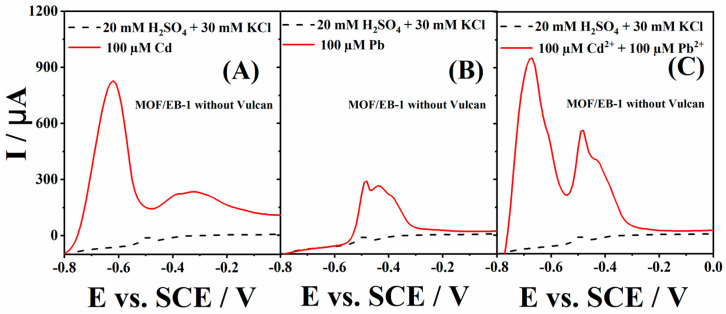
Voltammograms of MOF/EB-1 (without addition of Vulcan) electrode in 20 mM H_2_SO_4_ + 30 mM KCl (---) (**A**–**C**), 100 µM Cd^2+^ + 20 mM H_2_SO_4_ + 30 mM KCl (—) (**A**), 100 µM Pb^2+^ + 20 mM H_2_SO_4_ + 30 mM KCl (—) (**B**) and 100 µM Cd^2+^+ 100 µM Pb^2+^ +20 mM H_2_SO_4_ + 30 mM KCl (—) (**C**).

Voltammograms of pure MOF/EB-1 (without addition of Vulcan) obtained in the supporting electrolyte and 100 µM Cd^2+^ solution are presented in Figure 3A. An intense and well-defined peak of Cd oxidation to Cd^2+^ with the current of 0.827 mA at −0.62 V was noticed. An additional oxidation peak that appeared at about −0.35 V could be attributed to the hydrogen oxidation, as explained above [38]. In 100 µM Pb^2+^ solution, a peak current of 0.290 mA was recorded at −0.48 V, corresponding to the oxidation of Pb to Pb^2+^ at pure MOF/EB-1 (Figure 3B). Finally, the voltammogram of this electrode in 100 µM Cd^2+^ + Pb^2+^ solution (Figure 3C) revealed oxidation peaks with currents of 0.957 and 0.568 mA at potentials of −0.67 and −0.47 V, corresponding to the oxidation of Cd and Pb to Cd^2+^ and Pb^2+^ ions, respectively. It is worth noting that the signal intensity did not decrease in the solution containing both heavy metal ions, thus enabling their simultaneous sensing using pure MOF/EB-1 composite [3].

**Figure 4:**
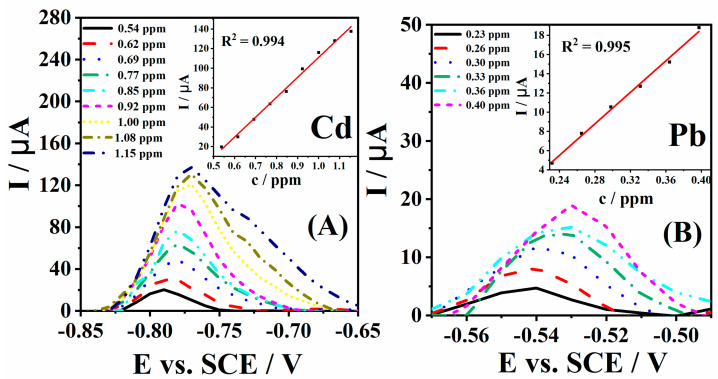
Voltammograms of the MOF/EB-1 electrode in 20 mM H_2_SO_4_ + 30 mM KCl solution with increasing concentrations of Cd^2+^ (**A**) and Pb^2+^ (**B**) with the corresponding standard addition plots in the inset.

The limits of detection (LOD) of Cd^2+^ and Pb^2+^ ions by the MOF/EB-1 electrode were determined using the three sigma method [43]. An amount of 0.077 and 0.033 ppm (0.1 µM) addition of Cd^2+^ and Pb^2+^ solutions were made for the supporting electrolyte (Figure 4). The standard addition plots presented in Figure 4A,B inset clearly show the linear range of 0.54–1.15 ppm (0.7 to 1.5 µM) for Cd^2+^ and 0.23–0.40 ppm (0.7–1.2 µM) for Pb^2+^. The lowest LODs of Cd^2+^ and Pb^2+^ ions of 0.077 ppm (0.103 µM) and 0.033 ppm (0.100 µM), respectively, were determined for the MOF/EB-1 electrode. The sensitivities of the MOF/EB-1 electrode for Cd^2+^ and Pb^2+^ ions, as a slope of corresponding standard addition plots presented in Figure 4A,B inset, were determined to be 0.203 mA ppm^−1^ (156 A M^−1^) and 0.082 mA ppm^−1^ (27 A M^−1^), respectively. PANI and polyaniline—poly (2,2-dithiodianiline), and PANI-PDTDA, [44] gave significantly higher LODs for Cd^2+^ and Pb^2+^ than here presented with the MOF/EB-1 electrode (Table 2). Amine-functionalized PANI mixed with exfoliated graphite oxide (EGAMPANI) showed activity for Cd^2+^ and Pb^2+^ detection with LOD of 1.2 and 0.98 µM, respectively [45]. Other polymers such as poly (3,4-ethylenedioxythiophene) (PEDOT) [46] gave a LOD of 0.6 and 0.5 ppm for Cd^2+^ and Pb^2+^, respectively. Chit-CNT film-modified glassy carbon electrode gave LODs of 0.6 and 0.8 ppm for Pb^2+^ and Cd^2+^ ions, respectively [38], with a linear range from 0.63 to 3.70 ppm for Pb^2+^ and 1.50 to 4.44 ppm for Cd^2+^. These values of LODs are about ten times higher than the LODs of two heavy metal ions sensing by the herein-tested electrode. The zeolitic imidazolate framework ZIF-8 synthesized with chitosan (CS) gave LOD of 0.135 µM for Cd^2+^ [47] which is again higher than the obtained LOD for Cd^2+^ ions presented here.

**Table 2:** Comparison of the performance of MOF/EB-1 electrode with other electrodes tested for the determination of Cd^2+^ and Pb^2+^ ions in the standard solutions.

Electrodes	Heavy Metal Ions	Linear Range	LOD	Ref.
MOF/EB-1	Cd^2+^	0.54–1.15 ppm (0.7 to 1.5 µM)	0.077 ppm (0.103 µM)	This work
	Pb^2+^	0.23–0.40 ppm (0.7 to 1.2 µM)	0.033 ppm (0.100 µM)	
EDTA_PANI/SWCNTs	Pb^2+^	-	0.34 ppm (1.65 µM)	[22]
PEDOT	Cd^2+^	5–20 ppm	0.6 ppm	[46]
	Pb^2+^	5–20 ppm	0.5 ppm	
EGAMPANI	Cd^2+^	-	0.15 ppm (1.2 µM)	[45]
	Pb^2+^	-	2.03 ppm (0.98 µM)	
PANI–PDTDA	Cd^2+^	1000–0.001 μM	0.29 μM	[44]
	Pb^2+^	1000–0.001 μM	0.17 μM	
PANI	Cd^2+^	1000–0.01 μM	0.86 μM	[44]
	Pb^2+^	1000–0.01 μM	1.3 μM	
Chit-CNT film	Cd^2+^	1.50–4.44 ppm	0.8 ppm	[38]
	Pb^2+^	0.63–3.70 ppm	0.6 ppm	
ZIF-8-CS	Cd^2+^	1.0–100 μM	0.048 ppm (0.135 µM)	[47]

**Figure 5:**
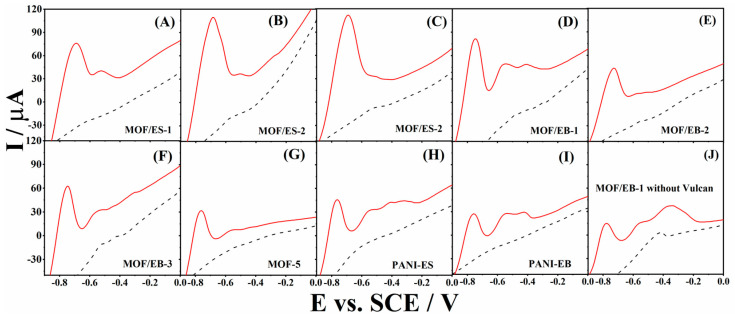
Voltammograms of MOF/ES-1 (**A**), MOF/ES-2 (**B**), MOF/ES-3 (**C**), MOF/EB-1 (**D**), MOF/EB-2 (**E**), MOF/EB-3 (**F**), MOF-5 (**G**), PANI-ES (**H**), PANI-EB (**I**), and MOF/EB-1 without Vulcan (**J**) in the sample from Danube River before (---) and after addition of Cd^2+^ and Pb^2+^ (—).

Simultaneous detection of Cd^2+^ and Pb^2+^ by all tested materials in a real sample, i.e., a sample from the Danube River without any pretreatment, is illustrated in voltammograms shown in Figure 5. It is evident that all composites are highly active for the detection of two heavy metal ions in the real sample. The peak current and potential values corresponding to the oxidation of Cd and Pb to Cd^2+^ and Pb^2+^, respectively, at the examined electrodes are summarized in Table 3. Interestingly, MOF/ES-3 and MOF/ES-2 electrodes gave the highest current of 0.111 and 0.110 mA due to Cd oxidation to Cd^2+^ at −0.69 V followed by the MOF/EB-1 electrode with an oxidation current of 0.081 mA at −0.75 V. On the other hand, the MOF/EB-1 electrode gave the highest current of 0.049 mA due to Pb oxidation to Pb^2+^ at −0.55 V, followed by the current of 0.037 mA at −0.50 V of the MOF/ES-2. The MOF-5 and MOF/EB-2 composites gave low oxidation currents during detection of both heavy metal ions. It is important to note that the pure MOF/EB-1 electrode (without the addition of Vulcan) also gave a good electrochemical response during the simultaneous oxidation of two heavy metals in the real sample. All obtained results assure that the herein examined composites could be applied as electroanalytical sensors for Cd^2+^ and Pb^2+^ ions detection.

## 4. Conclusions

Herein, six different composites of MOF-5 and PANI were thoroughly examined for the electrochemical detection of Cd^2+^ and Pb^2+^ ions in water samples by using the ASV technique. Comparative measurements with their starting components, MOF-5 and PANI, were also performed. A good electrochemical response of all composites to the presence of both heavy metal ions was observed. The MOF/EB-1 composite (71.0 wt.% MOF-5) gave the highest oxidation currents among all tested materials during both individual and simultaneous detection of Cd^2+^ and Pb^2+^ ions, overpassing the values of its individual components. Still, each individual component contributed to the composites’ response—MOF-5 providing high surface area for heavy metals adsorption during the accumulation step and PANI providing a network for electron transfer during the oxidation process. The most regular morphology, high specific surface area and good dispersibility manifested in its highest zeta potential are held responsible for the best performance of MOF/EB-1 for Cd^2+^ and Pb^2+^ ions sensing. Sensing of Cd^2+^ ions was not affected by the presence of Pb^2+^ unlike for many of reported electrochemical sensors for simultaneous heavy metal ion determination. Low limits of detection of 0.077 ppm and 0.033 ppm for Cd^2+^ and Pb^2+^, respectively, were attained using the MOF/EB-1 electrode. Finally, all six composites showed well-defined peaks of Cd and Pb oxidation to Cd^2+^ and Pb^2+^, respectively, in the real sample from the river (of higher currents compared to those recorded using their individual components), supporting their potential to be used as sensors for two heavy metals detection in water samples.

## Figures and Tables

**Table 1:** Values of zeta potential of studied materials (six MOF-5/PANI composites and their starting components) determined by DLS, and their oxidation peak current (I_p_) and peak potential (E_p_), measured during individual detection of Cd^2+^ and Pb^2+^ ions (Figure 1). Values of I_p_ and E_p_ for Vulcan are also given, for comparison.

Samples/Electrodes	Zeta Potential (mV)	Cd^2+^		Pb^2+^	
		I_p_/mA	E_p_/V	I_p_/mA	E_p_/V
MOF/ES-1	11.73	1.478	−0.62	0.574	−0.45
MOF/ES-2	9.06	1.361	−0.65	0.474	−0.45
MOF/ES-3	6.92	0.928	−0.65	0.286	−0.45
MOF/EB-1	14.93	2.235	−0.68	0.733	−0.46
MOF/EB-2	12.00	0.957	−0.64	0.263	−0.46
MOF/EB-3	11.77	1.765	−0.70	0.462	−0.48
MOF-5	−0.12	0.894	−0.67	0.251	−0.48
PANI-ES	13.14	1.358	−0.70	0.471	−0.48
PANI-EB	15.20	0.994	−0.70	0.479	−0.39
Vulcan	-	0.797	−0.57	0.647	−0.43

**Table 3:** The I_p_ and E_p_ of MOF-5/PANI composites, their starting components, and MOF/EB-1 composite electrode with no Vulcan added during simultaneous detection of Cd^2+^ and Pb^2+^ (Figure 3) in the real sample (water from the river Danube).

Electrodes	Cd^2+^		Pb^2+^	
	I_p_/mA	E_p_/V	I_p_/mA	E_p_/V
MOF/ES-1	0.076	−0.69	0.040	−0.52
MOF/ES-2	0.110	−0.69	0.037	−0.50
MOF/ES-3	0.111	−0.69	0.033	−0.51
MOF/EB-1	0.081	−0.75	0.049	−0.55
MOF/EB-2	0.043	−0.73	0.012	−0.57
MOF/EB-3	0.062	−0.75	0.032	−0.54
MOF-5	0.032	−0.76	0.007	−0.56
PANI-ES	0.046	−0.76	0.033	−0.55
PANI-EB	0.028	−0.76	0.027	−0.55
MOF/EB-1 without Vulcan	0.015	−0.78	0.015	−0.56

## Data Availability

The data that support the findings of this study are available on request from the corresponding author. The data are not publicly available due to privacy restrictions.

## Supplementary Materials

The following supporting information can be downloaded at: https://www.mdpi.com/article/10.3390/polym16050683/s1, Figure S1: Voltammograms of MOF/ES-1 (A), MOF/ES-2 (B), MOF/ES-3 (C), MOF/EB-1 (D), MOF/EB-2 (E), MOF/EB-3 (F), MOF-5 (G), PANI-ES (H), PANI-EB (I), and Vulcan (J) in 20 mM H_2_SO_4_ + 30 mM KCl supporting electrolyte (---) and in 100 µM Cd^2+^ solution (—); Figure S2: Voltammograms of MOF/ES-1 (A), MOF/ES-2 (B), MOF/ES-3 (C), MOF/EB-1 (D), MOF/EB-2 (E), MOF/EB-3 (F), MOF-5 (G), PANI-ES (H), PANI-EB (I), and Vulcan (J) in 20 mM H_2_SO_4_ + 30 mM KCl supporting electrolyte (---) and in 100 µM Pb^2+^ solution (—); Figure S3: Voltammograms of MOF/ES-1 (A), MOF/ES-2 (B), MOF/ES-3 (C), MOF/EB-1 (D), MOF/EB-2 (E), MOF/EB-3 (F), MOF-5 (G), PANI-ES (H), PANI-EB (I), and Vulcan (J) in 20 mM H_2_SO_4_ + 30 mM KCl supporting electrolyte (---) and in 100 µM Cd^2+^ and Pb^2+^ solution (—).
