# Supplementary material for: Electrochemical Sensing of Cadmium and Lead Ions in Water by MOF-5/PANI Composites
Source: Polymers (Basel). 2024 Mar 2;16(5):683. doi: 10.3390/polym16050683 (PMC10935052; doi:10.3390/polym16050683)
Supplement: Supplementary file 1 [file polymers-16-00683-s001.zip › polymers-2881598-supplementary.pdf]

Electrochemical sensing of cadmium and lead ions in water by MOF-5/PANI composites

Jadranka Milikić[a], Marjetka Savić[b], Aleksandra Janošević Ležaić[c], Biljana Šljukić[a*] and Gordana Ćirić-Marjanović[a]

[a] University of Belgrade, Faculty of Physical Chemistry, Studentski trg 12-16, 11158 Belgrade, Serbia

[b] University of Belgrade, Vinča Institute of Nuclear Science, National Institute of the Republic of Serbia, P.O. Box 522, 11001 Belgrade, Serbia

[c]University of Belgrade, Faculty of Pharmacy, Vojvode Stepe 450, 11221 Belgrade, Serbia

* corresponding author, E-mail: biljka@ffh.bg.ac.rs

Supplementary data

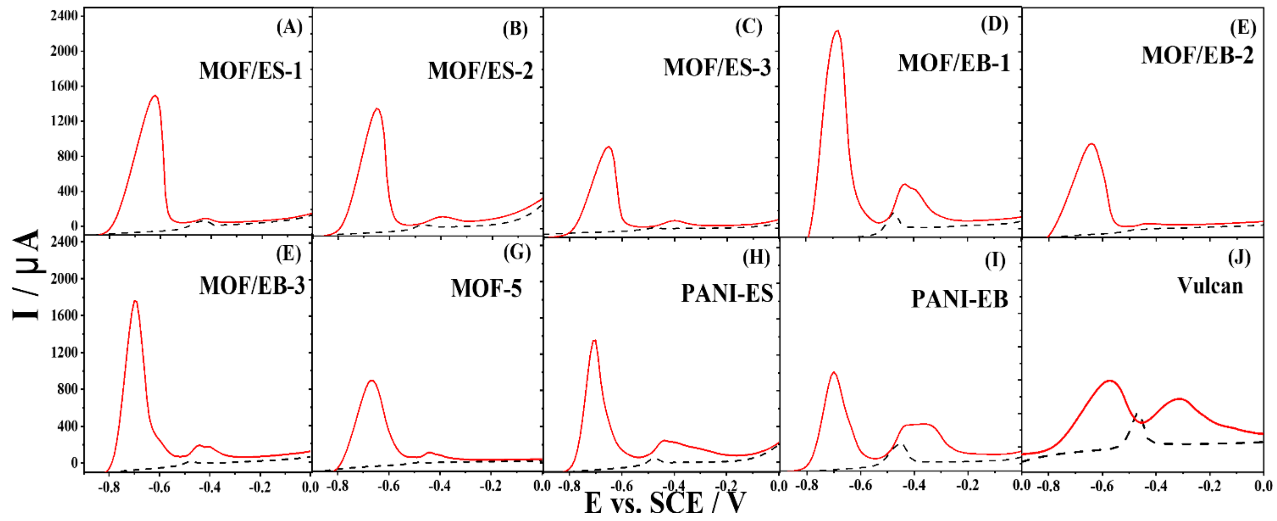

Figure S1. Voltammograms of MOF/ES-1 (A), MOF/ES-2 (B), MOF/ES-3 (C), MOF/EB-1 (D), MOF/EB-2 (E), MOF/EB-3 (F), MOF-5 (G), PANI-ES (H), PANI-EB (I), and Vulcan (J) in 20 mM H_2SO_4 + 30 mM KCl supporting electrolyte (---) and in 100 µM Cd^{2+} solution (–).

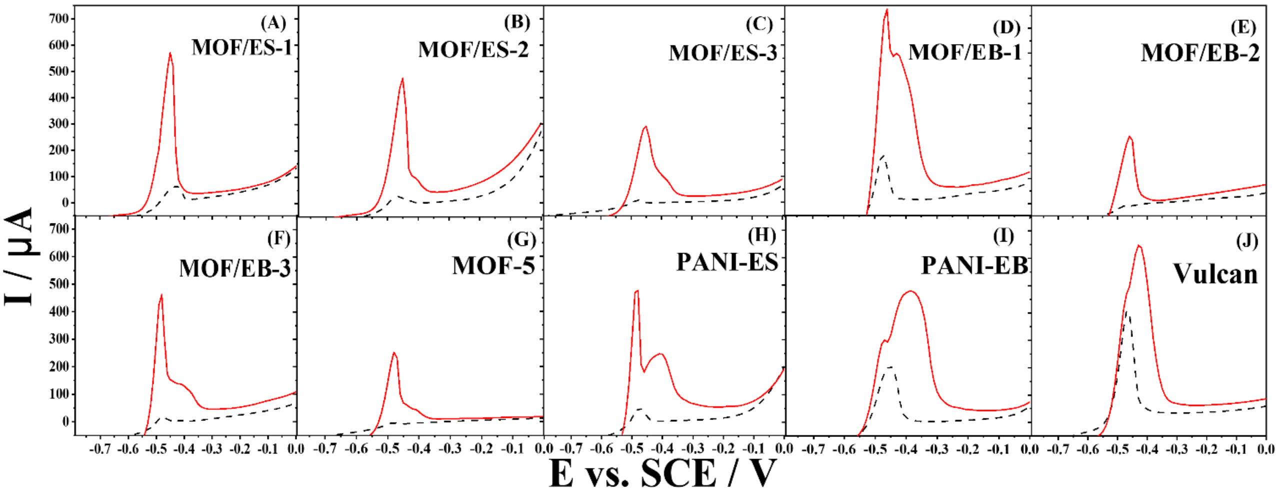

Figure S2. Voltammograms of MOF/ES-1 (A), MOF/ES-2 (B), MOF/ES-3 (C), MOF/EB-1 (D), MOF/EB-2 (E), MOF/EB-3 (F), MOF-5 (G), PANI-ES (H), PANI-EB (I), and Vulcan (J) in 20 mM H_2SO_4 + 30 mM KCl supporting electrolyte (---) and in 100 μM Pb^{2+} solution (—).

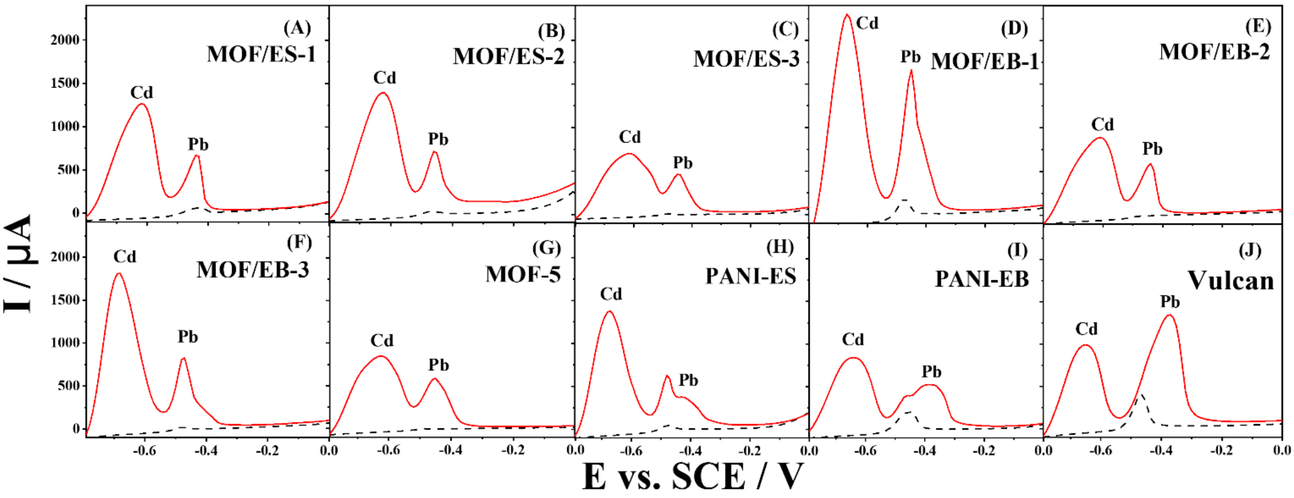

Figure S3. Voltammograms of MOF/ES-1 (A), MOF/ES-2 (B), MOF/ES-3 (C), MOF/EB-1 (D), MOF/EB-2 (E), MOF/EB-3 (F), MOF-5 (G), PANI-ES (H), PANI-EB (I), and Vulcan (J) in 20 mM H_2SO_4 + 30 mM KCl supporting electrolyte (---) and in 100 μM Cd^{2+} and Pb^{2+} solution (—).
